# Conservation of DNA-binding specificity and oligomerisation properties within the p53 family

**DOI:** 10.1186/1471-2164-10-628

**Published:** 2009-12-23

**Authors:** Tobias Brandt, Miriana Petrovich, Andreas C Joerger, Dmitry B Veprintsev

**Affiliations:** 1 MRC Centre for Protein Engineering, Cambridge, CB2 0QH, UK; 2 MRC Laboratory of Molecular Biology, Cambridge, CB2 0QH, UK

## Abstract

**Background:**

Transcription factors activate their target genes by binding to specific response elements. Many transcription factor families evolved from a common ancestor by gene duplication and subsequent divergent evolution. Members of the p53 family, which play key roles in cell-cycle control and development, share conserved DNA binding and oligomerisation domains but exhibit distinct functions. In this study, the molecular basis of the functional divergence of related transcription factors was investigated.

**Results:**

We characterised the DNA-binding specificity and oligomerisation properties of human p53, p63 and p73, as well as p53 from other organisms using novel biophysical approaches. All p53 family members bound DNA cooperatively as tetramers with high affinity. Despite structural differences in the oligomerisation domain, the dissociation constants of the tetramers was in the low nanomolar range for all family members, indicating that the strength of tetramerisation was evolutionarily conserved. However, small differences in the oligomerisation properties were observed, which may play a regulatory role. Intriguingly, the DNA-binding specificity of p53 family members was highly conserved even for evolutionarily distant species. Additionally, DNA recognition was only weakly affected by CpG methylation. Prediction of p53/p63/p73 binding sites in the genome showed almost complete overlap between the different homologs.

**Conclusion:**

Diversity of biological function of p53 family members is not reflected in differences in sequence-specific DNA binding. Hence, additional specificity factors must exist, which allowed the acquisition of novel functions during evolution while preserving original roles.

## Background

Sequence-specific transcription factors are responsible for processing environmental and developmental signals, and initiating the appropriate cellular response. The total number of transcription factors of an organism increases with its complexity: it is estimated to be around 300 for yeast, 1000 for worms and 3000 for humans [1]. Besides a DNA-binding domain, another common feature of many transcription factors, such as basic helix-loop-helix (bHLH) factors and basic-region leucine zipper (bZIP) factors, is an additional oligomerisation domain (OD) [2,3]. A functional role for oligomerisation is easy to rationalize: it combines the DNA-binding specificity of individual monomeric domains, leading to a substantial increase in binding affinity. Divergence of transcription factor function within a family could originate from evolutionary changes in the DNA-binding specificity and in the oligomerisation properties.

A highly important family of transcription factors that play a key role in cell-cycle control and development is that of p53, p63 and p73. p53 is at the centre of a tumour suppressor network [4,5], and, as such, is essential for the prevention of cancer [6,7]. Both p63 and p73 are involved in developmental processes. p63 is essential for epidermal morphogenesis and limb development, whereas p73 is involved in the development of neural structures and the pheromone detection system, among its other roles. Nevertheless, p63 and p73 are also involved in processes controlled by p53 [8]. Interestingly, different functions are also observed even for closely related p53 orthologs. For example, genes encoding proteins involved in DNA metabolism are responsive to p53 in humans but not in mice [9]. All three family members consist of a structured DNA-binding domain (DBD), an oligomerisation domain and intrinsically disordered N-terminal transactivation and C-terminal regulatory domains [10]. Additionally, p63 and p73 also contain a structured sterile alpha motif (SAM) and an inhibitory domain at the C-terminus [11]. The majority of cancer-associated p53 mutations are found in the DNA-binding domain [6,7], highlighting the importance of correct DNA recognition. p53 specifically binds to a 20 base pair (bp) consensus DNA sequence, also called a response element (RE), consisting of two repeats of 5'-RRRCWWGYYY-3' (where R = A or G; Y = C or T; W = A or T), separated by 0-13 bp [12,13]. In addition, p53 also recognises a large number of sequences that deviate from this consensus site definition [14,15]. Several studies have shown that p53, p63 and p73 can recognise the same sites [16-18]. Additionally, each protein has different isoforms [19], which, in most cases, have identical DNA-binding domains but exhibit differences in transcriptional activity, adding an additional layer of complexity [17].

Despite a high degree of sequence conservation, particularly in the DNA-binding and tetramerisation domains, p53, p63 and p73 fulfil at least partially different roles. The molecular basis of how closely related transcription factors differentiate between their respective target genes is only poorly understood. Here, we characterised the oligomerisation and DNA-binding properties of several p53 family members. Firstly, we determined the dissociation constants for dimers and tetramers of p53 family members using analytical ultracentrifugation. We then compared the DNA-binding specificity of full-length human p53 (Hsp53) with that of its paralogs p63 and p73, including the isoforms ΔNp63α, ΔNp63β, ΔNp63γ, ΔNp73β and an engineered truncated version of p73 containing DNA-binding and parts of the oligomerisation domain only (p73CT, residues 104-383). We also compared the DNA-binding specificity of human p53 with that of its orthologs from a number of species at varying evolutionary distances from humans: mouse (*Mus musculus*, Mmp53), frog (*Xenopus laevis*, Xlp53), zebrafish (*Danio rerio*, Drp53) and fruit fly (*Drosophila melanogaster*, Dmp53). In these measurements, we included effects of CpG methylation as an additional factor potentially influencing DNA-binding specificity. We used a method for quantification of DNA-binding specificity which we have recently developed [15,20]. Using fluorescence anisotropy titrations, we measured the effect of every possible single base pair substitution of a consensus sequence on the affinity of the proteins for DNA. The DNA-binding data were then used to identify putative binding sites within the human genome to assess the impact of the differences in DNA-binding specificity.

## Results

### Oligomerisation equilibria

We have shown previously that full-length human p53 dissociates into dimers at nanomolar concentration, and that oligomerisation is essential for high-affinity DNA binding [21,22]. Here, we studied the oligomerisation properties of members of the p53 family, namely Dmp53, Drp53, Hsp53, Mmp53, and Xlp53, as well as human ΔNp63β and ΔNp73β. The p63 and p73 isoforms contain intact DNA-binding and tetramerisation domains. We used sedimentation velocity analytical ultracentrifugation (SV-AUC) experiments with a fluorescence detection system [23], which allows measurements to be made at low nanomolar concentrations. To specifically incorporate a fluorophore, we expressed proteins with a C-terminal CCPGCC tetra-cysteine tag and labelled them with FlAsH-EDT2, an arsenic derivative of fluorescein [24].

**Figure 1:**
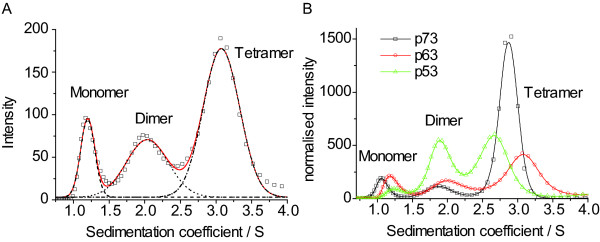
**Sedimentation profiles measured by fluorescence detection ultracentrifugation**. A) Exemplary fitting of data (ΔNp63β, 50 nM, squares) to three normal distributions indicated by dashed (monomer), dotted (dimer) and dash-dotted lines (tetramer). The overall fit is indicated by the red line. B: Comparison of sedimentation traces for human p53 (triangles, green line), ΔNp63β (circles, red line) and ΔNp73β (squares, black line) at a concentration of 50 nM (monomer). Intensities are normalised to the total integrated area.

The sedimentation profile of Hsp53 at 22.5 μM monomer concentration, measured using absorbance detection (data not shown), showed only one peak at ~2.9 S, which we assigned to a tetramer, because the protein has been shown to be tetrameric at this concentration [22]. Subsequently, we measured the sedimentation profiles of labelled proteins at different concentrations using the fluorescence detection system (Figure 1 and Additional file 1). At lower concentrations, a second peak appeared at 1.8 to 2.0 S. In order to improve the resolution of the sedimentation profiles in the range between 0.5 and 3 S, we repeated our experiments at higher rotor speeds (60 k rpm). In addition to the tetramer peak, we were able to resolve two peaks at 1.1 S and 1.9 S, which correspond to monomers and dimers, respectively.

All proteins studied formed tetramers which dissociate into dimers. For some proteins, these dimers dissociated into monomers. It was possible to determine their sedimentation profiles with well-resolved peaks, and thus to calculate the dissociation constants *K*_d _for the monomer-dimer and dimer-tetramer equilibria (Additional file 2, Figure 2). The dissociation constants for the dimer-tetramer equilibria of Dmp53, Drp53 and ΔNp73β were in the low nanomolar range. The self-association was about 5 times weaker for ΔNp63β and about 10 times weaker for Mmp53, Hsp53 and Xlp53. At low nanomolar concentrations, tetramers of human p53 dissociated into dimers, whereas those of human p63 and p73 readily dissociated into dimers and monomers. In the case of p63, the dissociation constants for momomer-dimer and dimer-tetramer equlibria were similar. For p73, the dimer-monomer *K*_d _was even larger than the dimer-tetramer *K*_d_. This indicates that only small amounts of p73 dimers are present in solution. For human p53, this is not the case, as the monomer-dimer *K*_d _is about 20 times lower than the dimer-tetramer *K*_d_. No monomers were observed for Dmp53 and Drp53.

**Figure 2:**
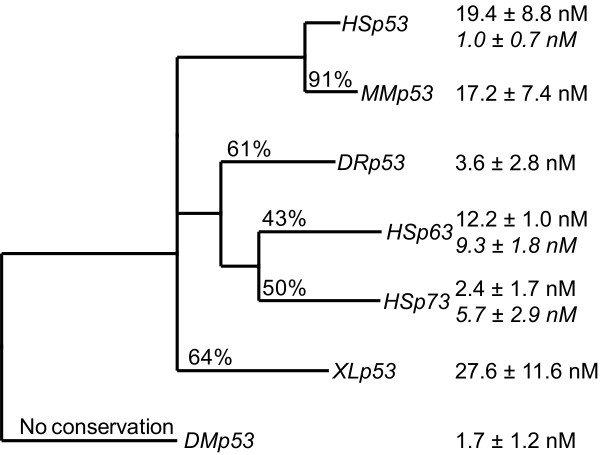
**Phylogenetic tree of the oligomerisation domain for selected members of the p53 family, with sequence similarity of the OD indicated on the branches**. Additionally, the *K*_d_s of the dimer-tetramer equilibrium and of the monomer-dimer equilibrium are given (italic numbers, expressed in concentration of monomer).

### DNA-binding specificity of p53 family members is highly conserved

It is often assumed that diverging transcription factors have differences in their DNA-binding specificity, which result in preferential recognition of a different response element sequence and an associated change in function. To answer the question of whether the p53 response element sequence is evolving and diverging, we compared the DNA-binding specificity of human p53, p63 and p73, and p53 from different species. We used a fluorescence anisotropy assay, which we had developed earlier for quantifying the DNA-binding specificity of Hsp53 [15,21].

**Figure 3:**
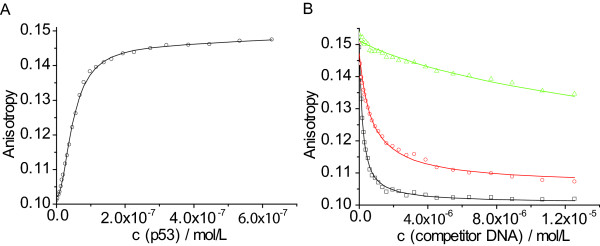
**Anisotropy titration assay**. A) Typical direct titration curve of a solution of 1.25 μ M Xlp53 titrated into 20 nM labelled Ref_Alexa488. Measured anisotropy values were fitted to a Hill equation allowing the calculation of the dissociation constant *K*_d _for the binding event between the labelled DNA and the protein. B) Displacement of a labelled reporter oligonucleotide from the complex by an unlabelled competitor oligonucleotide, reflected by a decrease in the anisotropy, allows accurate measurement of the difference in the *K*_d _between two sequences. Shown are typical titration curves for a competition experiment, in this case Xlp53: a 50 μM solution of competitor DNA was titrated into a solution of 20 nM labelled DNA and 400 nM Xlp53. Measured anisotropies are shown for a tight (Ref, log*K*_d _= -7.08, squares, straight line), an average (A5G, log*K*_d _= -6.64, circles, dashed line) and a weak (G7C, log*K*_d _= -5.70, triangles, dotted line) binding sequence with corresponding fits.

First, the *K*_d _between fluorescently labelled DNA and protein was measured using direct titrations (Figure 3A). Data were analysed using the Hill equation. The measured *K*_d _values were similar for all proteins studied (Table 1), and observed differences were within the error range of the method. The only exception was p73CT, which bound about 4-5 times more weakly than the ΔNp73β isoform. Weaker binding of p73CT can be attributed to impaired self-oligomerisation due to a truncation of the tetramerisation domain, which has been shown to destabilise the tetramer [25]. The Hill coefficient *n *was averaged over all measured datasets (*n *= 1.64), which was in close agreement with the value we have previously reported for human p53 [22]. In combination with analytical ultracentrifugation data, we can conclude that for all the proteins studied two dimers bind DNA cooperatively and form a tetramer, similarly to human p53 [21].

**Table 1:** DNA-binding parameters of p53 orthologs and paralogs.

Protein^a^	log*K*_*d *_(direct) GGACATGTCC^b,c^	log*K*_*d *_Ref (competition)^d^	Tightest binder log*K*_*d*_^e^	Consensus sequence using a Δlog*K*_*d *_ cut-off of 0.1 and 0.2 ^f^	Bit Score ^g^
Dmp53	-7.28 ± 0.03	-7.56	G**A**ACATGTCC -7.58	NRACATGTMB NDACRTGTHN	10.3

Drp53	-7.20 ± 0.03	-7.42	GG**G**CATG**C**CC -7.59	RRRCATGCCY VRRCATGYCB	13.1

Hsp53	-7.25 ± 0.03	-7.12	**A**GACATGTCC -7.14	RRRCWTGYCY NDRCWWGYCY	8.8

Mmp53	-7.02 ± 0.04	-6.88	GGACATG**C**CC -6.94	VRRCWTGYYY NDRCWWGYYN	6.2

Xlp53	-7.32 ± 0.02	-7.08	**A**GACATGTCC -7.13	VRRCATGYCY NDRCWWGYCY	10.9

ΔNp63α	-7.12 ± 0.01 (4)	-7.21	**A**G**G**CATGTCC -7.37	NDRCDWGYCH NNRCDDGYHN	7.2

ΔNp63β	-7.13 ± 0.04 (6)	-7.45	GG**G**CATGTCC -7.46	NRRCATGTCY NRRCRWGYCN	12.0

ΔNp63γ	-7.17 ± 0.02	-7.48	GGACATGTCC -7.48	NRRCATGTCB NRRCRWGYCN	12.7

ΔNp73β	-7.26 ± 0.02	-7.62	**C**G**G**CATGTC**T** -7.89	VRGCATGYCY NRGCAWGYCB	14.5

p73CT	-6.62 ± 0.04 (6)	-6.83	GG**G**CATGTCC -7.03	NRGCATGYCY NRRCAWGYCN	13.2

^a^Full-length proteins of p53 (Dm = *Drosophila melanogaster*, Dr = *Danio rerio*, Hs = *Homo sapiens*, Mm = *Mus musculus*, Xl = *Xenopus laevis*) and naturally occurring isoforms of human p63 and p73 were used. By contrast, p73CT is not naturally occurring and comprises p73 residues 104-383.

^b^Double stranded labelled reporter DNA (Alexa488-GGACATGTCCGGACATGTCCf)

^c^Each direct titration experiment was repeated 16 times, if not otherwise indicated after the standard deviation (SD) in brackets

^d^Double stranded unlabelled reference DNA GGACATGTCCGGACATGTCC

^e^Deviations from the reference sequence are shown in bold letters

^f^N = any nucleotide, D = A, G or T; R = G or A; M = A or C; B=G, C or T; H = A, C or T; W= A or T; Y = C or T; V = A, C or G

^g^For bit score explanation see figure 4.

**Figure 4:**
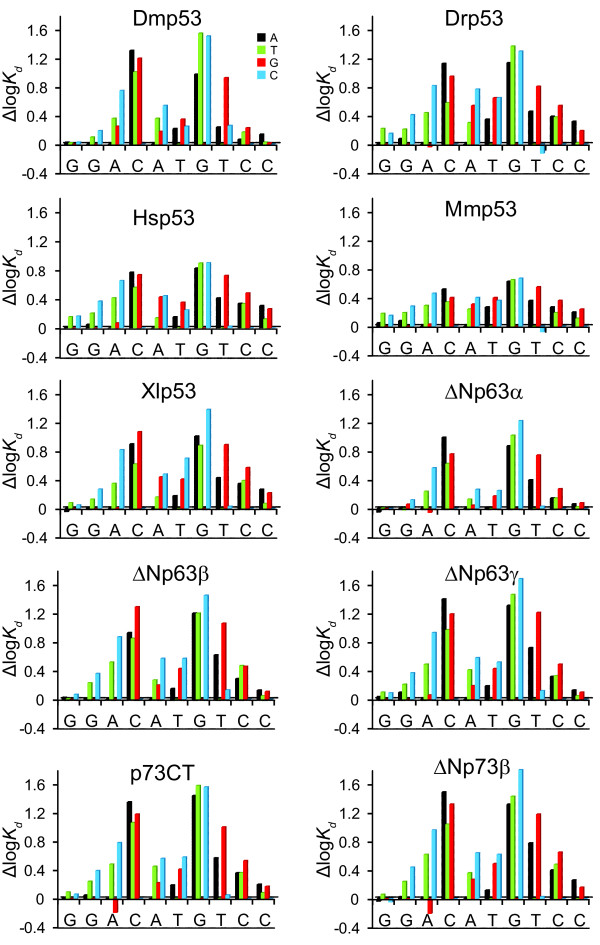
**DNA-binding specificity of p53 family members**. Δlog*K*_d _plot for all competitor DNA sequences. Affinity penalties with respect to the reference sequence caused by base pair substitutions are shown for all nucleotides (A = black, T = green, G = red, C = blue). The reference sequence is indicated below the axis. Only the analysed half-site is shown. A positive value indicates weaker binding of the competitor sequence than the reference sequence, whereas a negative value indicates that the substitution leads to tighter protein-DNA binding.

**Figure 5:**
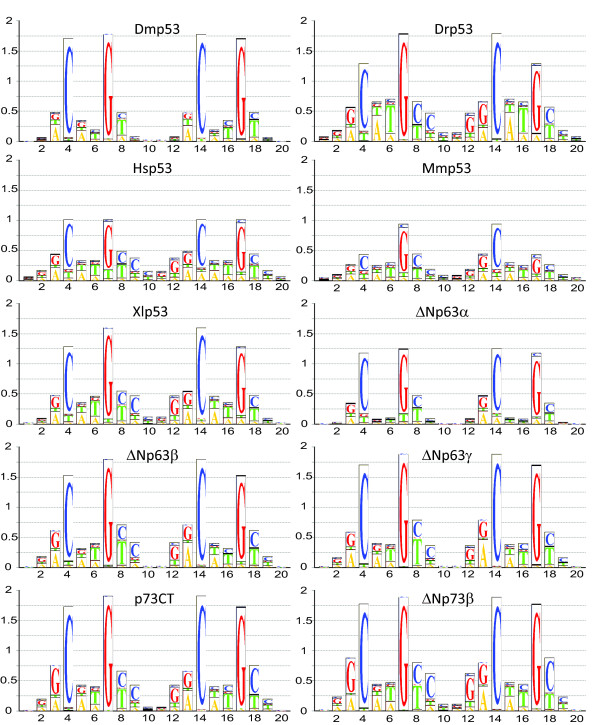
**Sequence logos for all p53 family proteins studied**. The complete response element is shown, and bit values are plotted against sequence position. A value of 0 means all four nucleotides bind with the same affinity and there is no selectivity, whereas the value 2 stands for absolute selectivity for one nucleotide, with the other three being highly penalised. A measure of the total information content (or selectivity) of the protein can be calculated by summing up all individual bit scores at every position. The maximum theoretical value of information content for a 20-bp response element is 40 bit.

The driving force for recognition of a specific DNA sequence surrounded by non-specific seqences is not the absolute affinity but rather specificity, or the relative affinity for specific vs. non-specific sequences. To define the DNA-binding specificity of the members of the p53 family, we measured the affinity of the proteins to all possible permutations of a reference consensus binding sequence (Additional file 3) using a fluorescence competition titration assay (Figure 3B). This sequence contains two identical copies of a GGACATGTCC half-site and is one of the tightest-binding sequences for human p53 [15]. The results for all the p53 homologs analysed are summarised in Figure 4. For every nucleotide substitution, the difference of the logarithm of the dissociation constants for the mutated sequence and the reference sequence (Δlog*K*_d_) was determined. High positive values of Δlog*K*_d _indicate high affinity penalties and low probability of observing this substitution in the binding site. The effects of nucleotide substitution are also presented as a sequence logo (Figure 5), which depicts the most preferred nucleotide at a position as the largest letter, and the relative selectivity at this position as the height of the bar. Based on the affinity differences, we calculated expected relative nucleotide frequencies for each position, and a corresponding bit score ranging from 0 to 2 [26,27]. Key features of the response element are highly conserved between all the proteins studied. The largest decrease in affinity was caused by nucleotide changes at positions 4 and 7, which correspond to the invariant C and G in the RRRCWWGYYY consensus sequence. Nucleotide changes at positions 5 and 6, corresponding to the central WW element, and positions 3 and 8, also caused significant changes in the affinity. Generally, changes at the outer positions 1, 2 and 9, 10 did not significantly affect binding. Accordingly, the largest contributions to the overall DNA-binding specificity are made by positions 4 and 7, followed by 3, 5, 6, and 8. The observed changes can be alternatively expressed as a consensus sequence definition (Table 1). Selecting the nucleotide changes resulting in the highest affinity at each position defines the highest affinity sequence. A better reflection of DNA-binding specificity is to apply a cut-off value representing the error of the measurement. All nucleotides at a particular position that cause a lower affinity change, Δlog*K*_d_, than the cut-off value are treated as having equal binding properties. Depending on the cut-off value, a number of different nucleotides can be present at a given position. For example, Dmp53 recognises the highest affinity sequence GAACATGTCC, which becomes NRACATGTMB at a cut-off value of 0.1 log*K*_d _units, and NDACRTGTHN at 0.2 log*K*_d _units, where N = any nucleotide; R = G or A; M = A or C; B = G, C or T; H = A, C or T; and W = A or T. As was shown for human p53 [14,15], the observed DNA-binding specificity for all the proteins studied is less stringent than the originally proposed definition of the p53 consensus sequence RRRCWWGYYY [12].

Despite the overall similarities of the DNA-specificity profiles, there are also some notable differences. The magnitude of the penalties with respect to the Δlog*K*_d _associated with nucleotide changes and the corresponding contribution to the overall specificity of binding varies for different proteins. Both mammalian (human and mouse) p53 proteins, which had the lowest bit score (Table 1), showed the lowest specificity. Evolutionarily more distant vertebrate proteins (zebrafish Drp53 and frog Xlp53) exhibited a selectivity pattern very similar to the mammalian proteins but showed higher bit score values of 10.8 and 12.8. Approximately 40 to 50% of the overall specificity came from positions 4 and 7. These positions were even more important for human p63 and p73 and invertebrate p53 (Dmp53), because they contributed 50 to 70% to the overall specificity. It is interesting to note that while most proteins prefered the C(A/T)(T/A)G motif at the centre of the half-site, p63, p73 and Dmp53 had a slight preference for G compared to T at position 5, recognising the motif C(A/G)(T/A)G or C(A/G/T)(T/A)G, depending on the selected cut-off. This observation resonates with findings of Osada et al. that p63 preferentially recognises RRRCGTGYYY [17], although A at position 5 resulted in stronger binding in our experiments. The other interesting feature is that p73 favoured G over A in position 3. This is in contrast to findings which suggest an A preceding the CWWG followed by a T forms the most stable complexes with p73 [18]. It is worth noting that the overall effects of nucleotide substitutions at positions 3 and 5 were relatively small compared to the effects at the positions 4 and 7.

While the isoforms ΔNp63β and ΔNp63γ behaved almost identically, the isoform ΔNp63α showed considerably smaller affinity penalties, meaning it is less specific. Interestingly, the DNA-binding affinities in the direct titrations and affinities for the reference sequence in competition experiments were similar for all isoforms. This suggests that the presence of the extreme C-terminal post-SAM domain in ΔNp63α may affect its DNA-binding specificity. Despite the significantly weaker binding of p73CT compared to ΔNp73β to DNA, the DNA-binding specificity of both p73 proteins was identical. This suggests that the DNA-binding specificity of tetrameric p73 is determined by the DNA-binding properties of individual DNA-binding domains, whereas the absolute affinity depends on the oligomerisation equilibrium.

### DNA methylation does not alter the specificity of p53 family members

CpG methylation has been shown to affect DNA recognition of transcription factors [28-30]. To investigate the effects of CpG methylation on DNA recognition of p53 family proteins, we used a method that we have previously applied to human p53 [20]. We systematically introduced a CpG dinucleotide at each position in the consensus p53 DNA binding sequence and identified substitutions tolerated by p53 family proteins. We then compared the binding affinities of methylated versus non-methylated sequences containing CpG (Additional file 4). Vertebrate p53 proteins (Mmp53, Xlp53 and Drp53) behaved similarly to human p53 and were mildly affected by substitutions at positions 2, 4 and 6. Interestingly, methylated sequences bound somewhat more tightly than non-methylated, although the effect of a single methylation was small. p63 and p73, along with invertebrate Dmp53, also tolerated CpG nucleotides at these positions. In particular, substitution at position 4 hardly changed the affinity, confirming that the CGTC central element of the binding site is recognised equally well as CATG, which is preferred by p53.

### Computational genome analysis

Transcription factors recognise a range of sequences which deviate from the highest affinity sequence. As a result of this deviation, the affinity of these sequences can be significantly weaker than that of the highest affinity sequence. We have previously shown that most of the reported p53 binding sites have affinity values up to 1.5 log*K*_d _units weaker than the highest affinity sequence, and that there is a very large number of potential binding sites in the genome [15]. In this study, the highest affinity sequence was practically identical for all the proteins studied, but the relative penalties for nucleotide substitutions were different. Such differential penalties may result in selection of non-overlapping sets of binding sites by different p53 family members.

**Figure 6:**
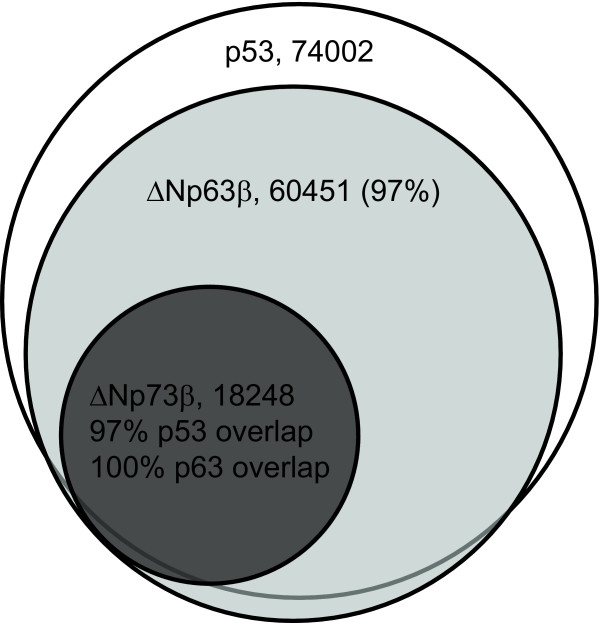
**Venn diagram of predicted p53, ΔNp63β and ΔNp73β sites in the human genome**.

To compare the selected sets of the putative binding sites, we computationally predicted all binding sites in the human genome using our affinity data (Additional file 5). We calculated affinity values for every position in the genome (see methods), and selected high-affinity ones using laboratory-developed software. Firstly, we compared the sets of binding sites predicted for human p53, p63 and p73 proteins (Figure 6 and Additional file 6). As we have shown previously for human p53 [15], the number of binding sites increases exponentially with an increasing cut-off value. Since the relative specificity of binding, as reflected by the bit-score value, is higher for p63 and p73 than for p53, there were fewer predicted sites selected at a cut-off value of 1.5 log*K*_d _units. We then determined the overlap between the predicted sets of binding sites, taking into account an error of prediction, *e*_p_, of 0.35 log*K*_d _units, which we had determined previously for Hsp53 [15]. For almost all proteins, the overlap was >98% at cut-off values between 0.5 and 1.5 Δlog*K*_d_. The only exception was Dmp53, which did not show overlap values higher than 68% with Hsp53. Remarkably, Dmp53 showed overlaps close to 100% with ΔNp63α. Overall, the results of computational analysis suggest that, based on DNA-binding preferences alone, all members of p53 family bind the same set of putative sites in the human genome. The observed quantitative differences in the binding preferences may result in different affinities toward specific binding site sequences, but not in diverging sets of target sites within a given genome.

## Discussion

### Oligomerisation properties of p53 family proteins

**Figure 7:**
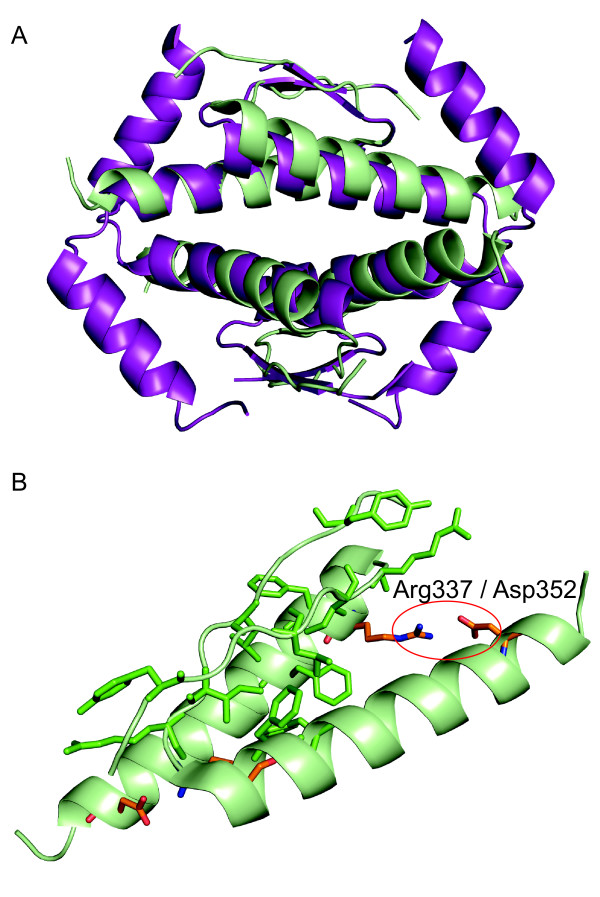
**The tetramerisation domain of human p53 and p73**. A) Superposition of the crystal structures of the tetramerisation domain of human p53 (green) [57] and human p73 (purple) [25], showing that p73 contains an additional C-terminal helix. This helix is conserved in the p63 sister protein. B) Primary-dimer interface of human p53. The side chains of Y327, L330, I332, R333, F338, L344 (green) and the salt bridge between R337 and D352 (orange) are shown as stick models. The salt bridge is not conserved in human p63 and p73.

The tetramerisation domain of Hsp53 (residues 325-356) is highly conserved in all vertebrate proteins of the p53 family [31]. A sequence alignment of the tetramerisation domain region of proteins used in this study is shown in Additional file 7. The Hsp53 tetramerisation domain forms a dimer of dimers and is composed of short monomeric building blocks consisting of a β-strand followed by an α-helix [32-34]. The primary dimers are stabilized by an intermolecular β-sheet and mainly hydrophobic helix packing interactions. In addition, the primary-dimer interface is stabilised by a salt bridge, which is typical for p53 orthologs but not found in its paralogs (Figure 7, Additional file 7). The tetrameric interface is formed by hydrophobic helix packing interactions. The hydrophobic interfaces are largely conserved in all the proteins studied except for Dmp53, which shows no significant sequence conservation and has a dimer-dimer interface that features a cluster of charged residues at its centre [31]. Importantly, recent structural studies have shown that the p73 tetramerisation domain contains an additional C-terminal helix, which is essential for the structural integrity and stability of the tetramer (Figure 7A). This helix is conserved in p63 and presumably has a similar structural role [25,35].

We determined dissociation constants for the monomer-dimer and dimer-tetramer equilibria of seven members of the p53 family (Figure 2, Additional file 2). Hsp53, Mmp53 and Xlp53 showed very similar *K*_d _values, consistent with the high conservation of contact residues. p63 and p73 form tighter tetramers than human p53, which, at least in the case of p73, can be attributed to extensive inter-dimer contacts made by the additional C-terminal helix (Figure 7A). Drp53, which, phylogenetically, can be placed somewhere between mammalian p53 and the p63/p73 paralogs [25], also forms more stable tetramers. What is most surprising is that Dmp53 forms tetramers with a comparable *K*_d_, while having a completely different dimer-dimer interface, suggesting that, despite structural divergence, the strength of the tetramer has been conserved through evolution.

Interestingly, the primary-dimer interface is tighter in p53 than in p73 (6-fold) and p63 (9-fold). Comparison of the Hsp53 and Drp53 sequences with p63 and p73 suggests that this difference in dimer stability may be attributed to the R337-D352 salt bridge that stabilizes the helix packing in the p53 primary dimer and large-to-small substitutions of hydrophobic residues in p63 and p73. The salt bridge is highly conserved in p53 across different species, and its disruption by a germline mutation (R337H) has been linked with adrenocortical carcinomas in children and other cancer forms [36,37]. p63 and p73 lack this intermolecular salt bridge and have a threonine (p63) and glutamine (p73) instead of the arginine in p53. As a result of the weakened dimer interface in p63 and p73, the dimers formed by tetramer dissociation are more likely to dissociate directly into monomers. Since key features of the primary dimer interface are highly conserved among different species for each paralog, it is likely that they exhibit dissociation equilibria similar to their human orthologs. The only exceptions are *Cavia porcellus *and *Pteropus vampyrus*, whose p53 lacks the paralog-specific salt bridge and may, therefore, also have weakened primary dimers. The observed differences in dissociation equilibria of the human paralogs may have important biological implications for interactions with regulatory proteins, such as members of the S100 family, which have been shown to differentially bind different oligomeric states of p53 [38,39]. Taken together, our results show that the overall strength of oligomerisation was conserved during the evolution of members of the p53 family, while subtle differences in the equilibria may play a role in fine-tuning their biological activity.

### DNA-contact residues are highly conserved in vertebrates

The sequence identity of the DNA-binding domain of p53 family members varies and is highest between p53 from closely related species, e.g. 86% identity between mouse and human proteins and ~60% between Drp53/Xlp53 and Hsp53. Hsp53 makes direct sequence-specific contacts with bases in the major groove of DNA via the side chains of K120, A276, C277 and R280. Contacts with the phosphate backbone are made by the side chains of S241, R248 and R273, and the backbone amides of K120 and A276 [40,41]. All DNA-contact residues are conserved in the vertebrate proteins studied (Additional file 8). Upon binding to a DNA half-site, two DBDs form a self-complementary protein-protein interface, mediated by residues P177, H178, R181, M243 and G244, which are conserved in vertebrate p53 [40,41]. In human p63 and p73 (~60% sequence identity with Hsp53), however, there are key substitutions in this region, indicating differences in the inter-DBD interactions. Dmp53 shows only 24% sequence identity to human p53 [42], with significant differences in the various DNA-binding motifs. K120 in the flexible L1 loop of Hsp5 binds to two purine bases in position 2 and 3 of the response element. The equivalent loop in Dmp53 is shortened and more rigid, making it unlikely that the lysine (K102 in Dmp53) forms the same DNA contacts as in Hsp53. In addition, the alanine (A276) making sequence-specific hydrophobic contacts in Hsp53 [40] is replaced by a threonine in Dmp53 (T262). Furthermore, the DNA-backbone contact residue R273 in Hsp53 is replaced by a lysine (K259). The L3 loop, which docks to the DNA minor groove via R248 in Hsp53, is also significantly different. It has a deletion and lacks the equivalent of R249, which plays a key role in stabilizing this region in Hsp53 [43]. Moreover, the L2/L3-loop region that forms the self-complementary DBD-DBD interface also shows variations, similarly to p63 and p73. Taken together, it would be reasonable to expect that the DNA-binding properties of Dmp53 differ from those of Hsp53.

### Conservation of the p53 response element and DNA-binding specificity

We quantified the DNA-binding properties of several members of the p53 family and investigated their ability to recognise methylated DNA. We found that the DNA-binding specificity of both orthologs and paralogs of p53 was conserved. Human and mouse p53 proteins showed almost identical specificity, consistent with their highest sequence conservation. It is also interesting to note that they exhibited the lowest absolute specificity, as reflected by the lowest bit score of the derived motif. Evolutionarily more distant vertebrate p53 proteins (Xlp53 and Drp53) showed a very similar specificity profile but somewhat higher specificity. There seems to be a very interesting underlying correlation: the more complex the organism and the more complex the p53 pathway, the lower the absolute specificity. p63 and p73 showed slightly different DNA-binding specificity compared with p53. This difference may be the result of the different residues in p63 and p73 being responsible for the interaction between two DBDs upon binding to a half-site motif. Despite the low sequence similarity of Dmp53 and human p53, and their aforementioned differences in key DNA-binding motifs, the DNA-binding specificity of Dmp53 is preserved and is similar to that of vertebrate p53 family members, in particular the more ancestral p63 and p73 proteins. The longest p63 isoform tested, ΔNp63α, has a significantly reduced DNA-binding specificity compared to other isoforms. It is possible that the additional post-SAM domain present in this isoform is directly or indirectly involved in regulation of its sequence-specific binding.

Using the affinity prediction, we identified all putative binding sites in the human genome for p53, p63 and p73 proteins. Despite quantitative differences in their DNA-binding specificity, all transcription factors studied select overlapping sets of binding sites. We found many more putative binding sites than have been previously identified in genome-wide experiments for p53/p63/p73 proteins [44-46]. The vast majority (95%) of experimentally identified p53 binding sites [44] contains a site predicted using our affinity data. The published dataset for p63 [45] consists of 5000 sites, which is significantly more than the 1700 sites reported for p53. Less than 20% of these 5000 sites contain a predicted high-affinity p63 site within a 500 bp window, perhaps reflecting different stringency criteria in peak calling in these two studies. Despite these differences, analysis of all *in vivo *binding-site sequences in these studies generated positional weight matrices, represented as sequence logos, which are very similar to the sequence logos derived by us based on *in vitro *binding affinity. This strongly suggests that the driving force for localisation of p53/p63/p73 to their respective sites in the genome is their sequence-specific binding. A recent study using a novel microsphere assay showed that the DNA-binding specificity of endogenous p53 in cell lysate is the same as that of the purified recombinant p53 from our work [47]. Nevertheless, several validated p53 response elements contain non-canonical sequences [48,49]. It was shown, that p53 acts weakly to moderately on response elements that contain only a half or a three quarter site of the canonical consensus sequence [50]. This is in accordance with our results, as we observed considerable binding to DNA with a mutated quarter or half site, which de facto represents a non-canonical p53 response element. Binding to non-canonical response elements may be facilitated by co-activating transcription factors. A comprehensive comparison between *in vivo *and *in vitro *binding can be found in an excellent recent review [51].

How can transcription factors with virtually identical DNA-binding specificity elicit different biological responses? There is also the closely related question of how transcription factors select their binding site in the genome, among many potential sites of comparable affinity? The "chromatin structure" and "DNA accessibility" concepts may at least partially answer the second question, although the mechanism controlling the chromatin structure with the specificity required is presently unknown. Different expression patterns of transcription factors and/or their abundance in the nucleus can also contribute to their specificity. The involvement of additional specificity factors would answer both questions. Such additional specificity factors should also bind DNA in a sequence-specific manner, and are likely to be transcription factors.

## Conclusions

Taken together, our data show that tetramerisation of p53 family members, which is important for high-affinity DNA binding, was established very early in the evolution of the p53 family and has been functionally conserved ever since. Despite significant differences in the contact surfaces involved, the strength of oligomerisation was preserved. Intriguingly, the DNA-binding specificity of different p53 family members is highly conserved even for evolutionarily distant species. This suggests that original functions were preserved while new functions were acquired during evolution, utilising the same DNA-binding specificity. The "core function" DNA-binding specificity of the p53 transcription factor network did not substantially change during evolution. Instead, there is accumulating evidence that functional divergence of the p53 family evolved through changes in the connectivity within the network, for example by interactions of p53 family members with different sets of co-activating transcription factors.

## Methods

### Protein cloning

For human full-length p53 we used wild type protein for DNA-binding experiments and a super-stable mutant, which has four mutations in the core domain (QM-Hsp53, M133L/V203A/N239Y/N268D) [52,53], for analytical ultracentrifugation experiments. A plasmid encoding Mmp53 was kindly provided by Geoffrey Wahl. Dmp53 was amplified from a cDNA library kindly provided by Simon Bullock. Coding sequences encoding for other studied proteins were amplified from clones obtained from the Mammalian Gene Collection (MGC), distributed via Geneservice (UK). For the ΔNp63γ isoform, parts of the gene were amplified from a genomic DNA library (Geneservice). Additionally, we made a p73 construct containing the DBD and parts of the OD (p73CT, residues 104-383). All inserts were subcloned into a pET24a-HLTEV plasmid containing an N-terminal 6xHis purification tag, a lipoyl domain [54] for improved solubility and a TEV-protease cleavage site. Constructs containing a C-terminal FlAsH-tag CCPGCC [24] were designed in a similar manner.

### Small scale expression screening

Small-scale screening for soluble expression in different cell lines was performed in 2 ml cultures on microplates in 2xTY media following induction with 1 mM IPTG. Proteins were purified using His-Fusion magnetic beads (BioClone Inc) on a BioSprint15 robot (Qiagen). Purified fractions were analysed by SDS-PAGE pre- and post-digestion with TEV-protease.

### Expression and purification

Large-scale expression and purification was carried out largely as described earlier [20,22]. All proteins were overexpressed in *E. coli *BL21 or B834 cells (Novagen) at 18°C for 16-20 h and purified using standard Ni-affinity chromatography protocols. Subsequently, the N-terminal tags were cleaved off by TEV-protease digestion. As a second purification step for p53 orthologs, heparin affinity chromatography was used. Solutions were diluted to reduce the salt concentration to about 30 mM NaCl. Proteins were eluted using a 20 column volume NaCl gradient (0 to 1 M NaCl). The final purification step was gel filtration chromatography using a Superdex 200 16/60 preparative gel filtration column (GE Healthcare) in 225 mM NaCl, 25 mM sodium phosphate pH 7.2, 10% glycerol and 5 mM DTT. Protein purity of >95% was determined by SDS-gel electrophoresis. Samples were flash frozen in liquid nitrogen and stored at -80°C until used.

### Labelling proteins with FlAsH

Labelling of C-terminally FlAsH-tagged (CCPGCC) proteins [24] was performed in 150 mM NaCl, 25 mM phosphate (pH 7.2), 10% glycerol, and 1 mM β-mercaptoethanol. 200 μL of 10 μM FlAsH-tagged protein were incubated with 1.5 equivalents of FlAsH-EDT_2 _(Lumio Green, Invitrogen) at 8°C for 2.5 h. We estimated that the stock solution was supplied at a concentration of approximately 1 mM. Excess label was removed by dialysis into the above buffer. Labelled proteins could be frozen and stored for at least a few months. The labelling reaction could easily be reversed by adding DTT, so care had to be taken to avoid DTT in buffers.

### Sedimentation velocity experiments

We used a XL-I analytical ultracentrifuge (Beckman) equipped with an AVIV fluorescence detection system (AVIV Biomedical). Experiments with C-terminally FlAsH-tagged proteins and unlabelled QM-Hsp53 (using an absorbance detection system) were done in 150 mM NaCl, 25 mM phosphate (pH 7.2), 10% glycerol, BSA (0.2 mg/mL) and 1 mM β-mercaptoethanol at 10°C. For fluorescence measurements, cells were pre-treated with a concentrated (1 mg/ml) solution of BSA and allowed to dry before loading samples. Sample volume was 80-90 μL at concentrations of 5-500 nM in SedVel60K fluorescence velocity cells (Spin Analytical). At least 15 measurements were done for each protein. Buffer density and viscosity were calculated using SEDNTERP software. Data analysis to obtain sedimentation coefficient traces was done with SEDFIT software [55]. Since only the tetramer peak at 3 S was detected in experiments with Hsp53 without the FlAsH-tag, we ignored peaks at higher sedimentation coefficients found for FlAsH-tagged proteins as artefacts caused by cross-linking of oxidised cysteines of the tag. Fitting of sedimentation profiles to normal distributions and *K*_d _calculation was done with our own laboratory software to estimate the relative amount of dimers and tetramers. The reported values for human p53 are somewhat lower than the values we have reported previously [56]. Most likely, a change in the cell design resulting in significantly lower surface area of exposed epoxy material and pre-treatment of the cells with concentrated BSA solution minimised the adsorption of p53 proteins to the cell wall, thereby increasing the fraction of material present in solution.

### Fluorescence anisotropy spectroscopy

All experiments were carried out in 96-well plates using a Pherastar plate reader (BMG Labtech) equipped with a Bravo 96-channel pipetting robot (Velocity 11) as previously described [15]. Buffer conditions for all experiments were 25 mM NaPi, 225 mM NaCl, 10% v/v glycerol, 5 mM DTT and 0.2 mg/mL BSA. Titrations were done at 22°C and repeated at least three times. Direct titrations were done as previously described [22] using 20 nM 5'-Alexa488-GGACATGTCCGGACATGTCC labelled DNA (Operon). The stock solution of 1.25 μM protein was titrated in small amounts, which allows calculation of the *K*_d _for the binding of labelled DNA to protein [21]. For competition experiments, a mixture of protein (at a concentration four times above the *K*_d _value, measured by direct titrations) and 20 nM labelled DNA were used as analytes, and competitor DNA (50 μM) was titrated in small steps. Over 3000 titrations were performed in total. Data were analysed according to cooperative binding and competition models using laboratory developed software [15].

### Computational search for putative binding sites

The putative binding sites in the genome were located using p53BindingSite software [15], available at http://www.mrc-lmb.cam.ac.uk/dbv. In short, the DNA-binding affinity was predicted for each position in the genome using binding affinity positional matrices measured for each protein studied, and positions with predicted affinity higher than the cut-off value were selected. We used human genome release 36.3, zebrafish genome release 10/06/2008 (International Human Genome Sequencing Consortium), fruit fly genome release 5 (The FlyBase Consortium/Berkeley Drosophila Genome Project) and mouse genome release 37 (Mouse Genome Sequencing Consortium). Instead of *Xenopus laevis *we used the *Xenopus tropicalis *genome (release 4.1, DOE Joint Genome Institute), as it is complete. We set the gap between both half-sites of the RE to be 0 and 1.

## Authors' contributions

DBV conceived research; TB, MP and DBV performed experiments, TB, MP, ACJ and DBV analysed and interpreted the results; TB and MP prepared figures; TB, ACJ and DBV wrote the manuscript. All authors read and approved the final manuscript.

## Supplementary Material

Additional file 1 **Figure S1**. Example of raw fluorescence data from analytical ultracentrifugation experiments. Click here for file

Additional file 2 **Table S1**. Summary of the calculated dissociation constants, observed sedimentation coefficients and protein concentration ranges used in analytical ultracentrifugation experiments. Click here for file

Additional file 3 **Table S2**. DNA sequences used for anisotropy experiments. Click here for file

Additional file 4 **Figure S2**. Results of fluorescence anisotropy binding assays using methylated DNA. Click here for file

Additional file 5 **Table S3**. DNA binding specificity positional weight matrices for p53 family members. Click here for file

Additional file 6 **Table S4**. Results of computational prediction of putative binding sites within the genome. Click here for file

Additional file 7 **Figure S3**. Sequence alignment of the tetramerisation domains of the p53 family members. Click here for file

Additional file 8 **Figure S4**. Sequence alignment of the DNA-binding domains of the p53 family members. Click here for file
