# Supplementary material for: Conservation of DNA-binding specificity and oligomerisation properties within the p53 family
Source: BMC Genomics. 2009 Dec 23;10:628. doi: 10.1186/1471-2164-10-628 (PMC2807882; doi:10.1186/1471-2164-10-628)
Supplement: Additional file 1 — Figure S1. Example of raw fluorescence data from analytical ultracentrifugation experiments. [file 1471-2164-10-628-S1.PDF]

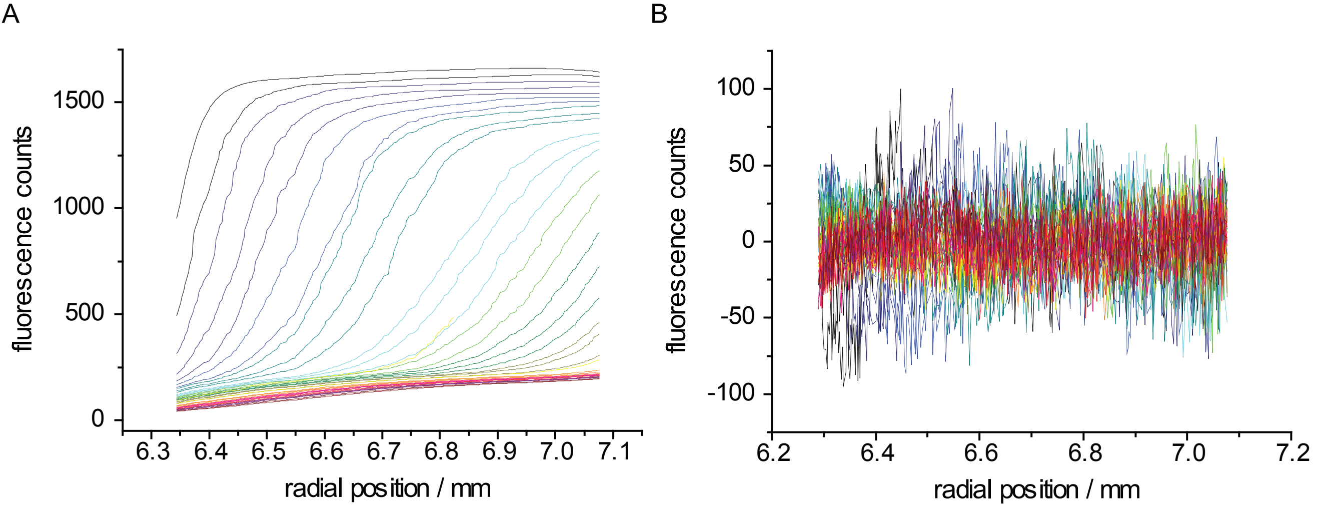

Figure S1. Raw data of analytical ultracentrifugation experiments. A) Traces of fluorescence measurements as function of radial position. The first scans are shown in black, the last scans are shown in red. B) Residual error for fit traces.
