# Supplementary material for: Conservation of DNA-binding specificity and oligomerisation properties within the p53 family
Source: BMC Genomics. 2009 Dec 23;10:628. doi: 10.1186/1471-2164-10-628 (PMC2807882; doi:10.1186/1471-2164-10-628)
Supplement: Additional file 2 — Table S1. Summary of the calculated dissociation constants, observed sedimentation coefficients and protein concentration ranges used in analytical ultracentrifugation experiments. [file 1471-2164-10-628-S2.PDF]

Table S1. Self-oligomerisation of p53 family members studied by fluorescence detection SV-AUC experiments.

Protein-FlAsH[a]	K_d ± SD (nM)		Concentration range monomer (nM)	Sedimentation coefficient (S)		
	Dimer-Tetramer	Monomer-Dimer		Tetramer	Dimer	Monomer [b]
Dmp53	1.7 ± 1.2	-	5 – 50	2.82	1.77	n.d.
Drp53	3.6 ± 2.8	-	5 – 100	2.96	1.82	n.d.
QM-Hsp53	19.4 ± 8.8	1.0 ± 0.7	20 – 50	2.75	1.93	1.22
Mmp53	17.2 ± 7.4	-	25 – 62.5	3.11	1.95	n.m.
Xlp53	27.6 ± 11.6	-	20 – 500	2.91	1.89	n.m.
ΔNp63β	12.2 ± 1.0	9.3 ± 1.8	50	3.02	1.88	1.04
ΔNp73β	2.4 ± 1.7	5.7 ± 2.9	25 - 50	2.85	1.96	1.18

[a]Full-length proteins of p53 (Dm = *Drosophila melanogaster*, Dr = *Danio rerio*, QM-Hs = quadruple mutant [1] *Homo sapiens*, Mm = *Mus musculus*, Xl = *Xenopus laevis*) and naturally occurring isoforms of human p63 and p73 were used.

[b]n.d.: not detectable; n.m.: not measured

1. Nikolova PV, Henckel J, Lane DP, Fersht AR: **Semirational design of active tumor suppressor p53 DNA binding domain with enhanced stability**. *Proc Natl Acad Sci U S A* 1998, **95**(25):14675-14680.
