# Supplementary material for: Conservation of DNA-binding specificity and oligomerisation properties within the p53 family
Source: BMC Genomics. 2009 Dec 23;10:628. doi: 10.1186/1471-2164-10-628 (PMC2807882; doi:10.1186/1471-2164-10-628)
Supplement: Additional file 3 — Table S2. DNA sequences used for anisotropy experiments. [file 1471-2164-10-628-S3.PDF]

Table S3. DNA sequences used for competition experiments.

Sequence name	Sequence*
Ref (reference sequence)	CGCGGACATGTCCGGACATGTCCCGC
G1A	CGC***A***GACATGTCCGGACATGTCCCGC
G1T	CGC***T***GACATGTCCGGACATGTCCCGC
G1C	CGC***C***GACATGTCCGGACATGTCCCGC
G2A	CGCG***A***ACATGTCCGGACATGTCCCGC
G2T	CGCG***T***ACATGTCCGGACATGTCCCGC
G2C	CGCG***C***ACATGTCCGGACATGTCCCGC
A3T	CGCGG***T***CATGTCCGGACATGTCCCGC
A3G	CGCGG***G***CATGTCCGGACATGTCCCGC
A3C	CGCGG***C***CATGTCCGGACATGTCCCGC
C4A	CGCGGA***A***ATGTCCGGACATGTCCCGC
C4T	CGCGGA***T***ATGTCCGGACATGTCCCGC
C4G	CGCGGA***G***ATGTCCGGACATGTCCCGC
A5T	CGCGGAC***T***TGTCCGGACATGTCCCGC
A5G	CGCGGAC***G***TGTCCGGACATGTCCCGC
A5C	CGCGGAC***C***TGTCCGGACATGTCCCGC
T6A	CGCGGACA***A***GTCCGGACATGTCCCGC
T6G	CGCGGACA***G***GTCCGGACATGTCCCGC
T6C	CGCGGACA***C***GTCCGGACATGTCCCGC
G7A	CGCGGACAT***A***TCCGGACATGTCCCGC
G7T	CGCGGACAT***T***TCCGGACATGTCCCGC
G7C	CGCGGACAT***C***TCCGGACATGTCCCGC
T8A	CGCGGACATG***A***CCGGACATGTCCCGC
T8G	CGCGGACATG***G***CCGGACATGTCCCGC
T8C	CGCGGACATG***C***CCGGACATGTCCCGC
C9A	CGCGGACATGT***A***CGGACATGTCCCGC
C9T	CGCGGACATGT***T***CGGACATGTCCCGC
C9G	CGCGGACATGT***G***CGGACATGTCCCGC
C10A	CGCGGACATGTC***A***GGACATGTCCCGC
C10T	CGCGGACATGTC***T***GGACATGTCCCGC
C10G	CGCGGACATGTC***G***GGACATGTCCCGC

*All sequences are flanked by CGC to ensure better annealing properties.
