# Supplementary material for: Conservation of DNA-binding specificity and oligomerisation properties within the p53 family
Source: BMC Genomics. 2009 Dec 23;10:628. doi: 10.1186/1471-2164-10-628 (PMC2807882; doi:10.1186/1471-2164-10-628)
Supplement: Additional file 4 — Figure S2. Results of fluorescence anisotropy binding assays using methylated DNA. [file 1471-2164-10-628-S4.PDF]

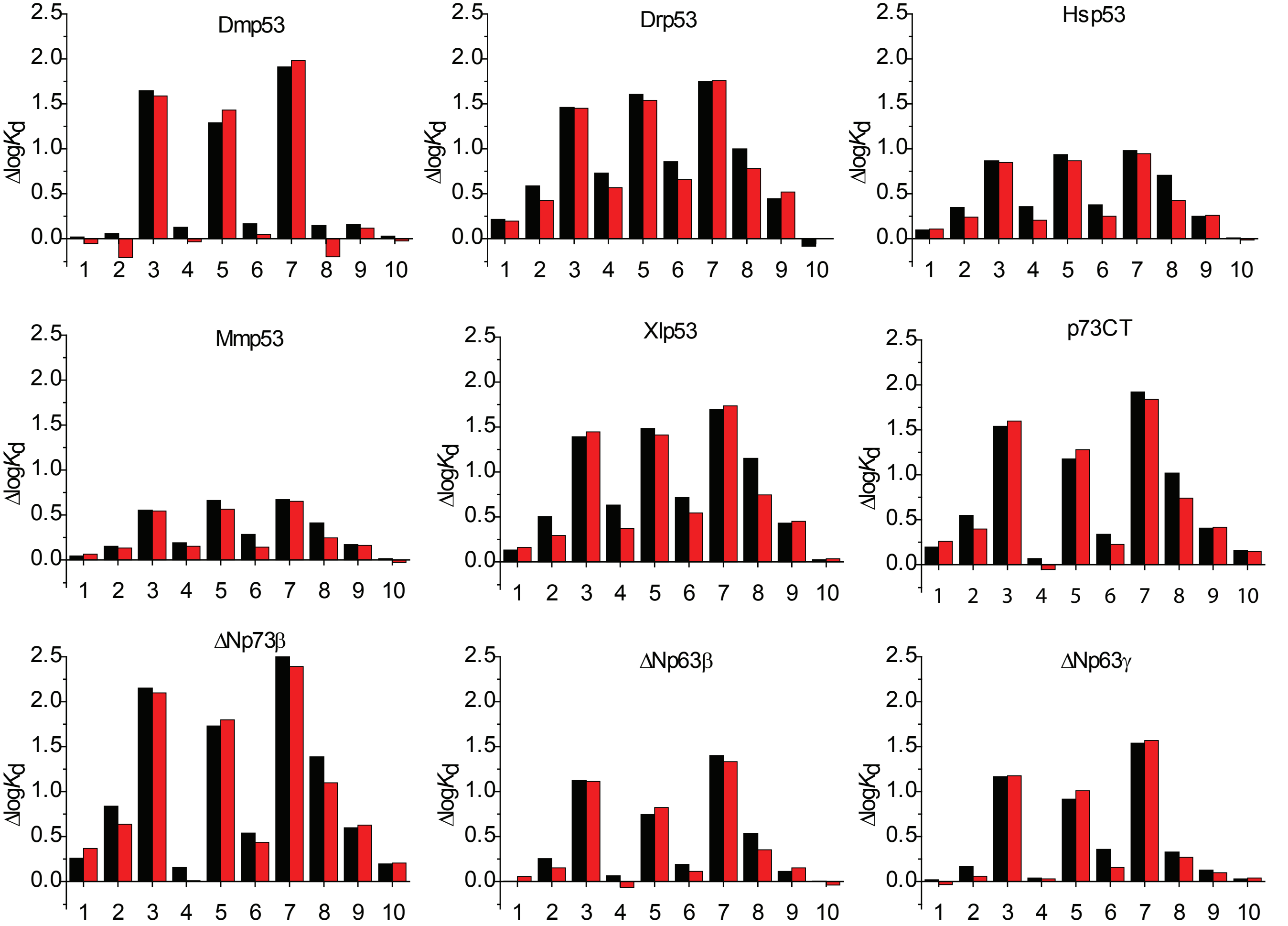

Figure S2. Results of fluorescence anisotropy experiments with methylated DNA. CpG (black bars) or 5-methyl-CpG (red bars) dinucleotide was systematically substituted into the reference sequence at position 1-10 of the first half site. The CpG position refers to the position of the C in the first half of the p53 binding sequence GGACAT-GTCC. The difference in affinity relative to the unmethylated non-methylated reference sequence is shown (ΔlogKd = logKd(i) - logKd(ref)). The logKd of the reference sequence GGACATGTCCGGACATGTCC, flanked by CGC triplets was -7.55. For more details of the method see reference [20].
