# Supplementary material for: Conservation of DNA-binding specificity and oligomerisation properties within the p53 family
Source: BMC Genomics. 2009 Dec 23;10:628. doi: 10.1186/1471-2164-10-628 (PMC2807882; doi:10.1186/1471-2164-10-628)
Supplement: Additional file 6 — Table S4. Results of computational prediction of putative binding sites within the genome. [file 1471-2164-10-628-S6.PDF]

Table S4. Sets of putative binding sites of p53 family members in the genome predicted using our affinity data.

Protein[a]	DNA[b]	Cut-off above K_d				**bp / bs**	Overlap with Hsp53		
		cutoff 0.5	1	1.5	2	**$*10^4$**	cut-off 0.5 / $0.5+e_p$	cut-off 1.0 / $1.0+e_p$	cut-off 1.5 / $1.5+e_p$
Dmp53	Dm	28	2642	23625	132104	**6.8**	**---**	**---**	**---**
	Hs	798	31735	264452	1377649	**10**	**13 / 55**	**40 / 68**	**51 / 68**
							**15 / 81** [c]	**68 / 95** [c]	**86 / 99** [c]
Drp53	Dr	25	510	5531	47856	**330**	---	---	---
	Hs	138	1578	15291	110938	**210**	68 / 100	80 / 100	96 / 100
Hsp53	Hs	172	4346	74002	695373	**76**	---	---	---
Mmp53	Mm	577	7716	151408	---	**34**	---	---	---
	Hs	123	5861	144467	---	**56**	55 / 100	62 / 97	73 / 99
Xlp53	Xt	108	3057	46717	342778	**56**	---	---	---
	Hs	431	8461	97211	631454	**39**	97 / 100	87 / 98	76 / 96
p73CT	Hs	60	2135	21409	143797	**150**	52 / 97	58 / 98	75 / 98
ΔNp73β	Hs	164	2325	18248	91998	**140**	36 / 90	62 / 94	80 / 99
ΔNp63α	Hs	4389	191391	1463912	9415161	**1.7**	98 / 100	88 / 92	82 / 97
ΔNp63β	Hs	283	5593	60451	338048	**59**	70 / 99	62 / 93	51 / 95
ΔNp63γ	Hs	89	2829	38336	200551	**120**	72 / 98	64 / 98	69 / 98

[a] Full-length proteins of p53 (Dm = *Drosophila melanogaster*, Dr = *Danio rerio*, Hs = *Homo sapiens*, Mm = *Mus musculus*, Xl = *Xenopus laevis*) and naturally occurring isoforms of human p63 and p73 were used. By contrast, p73CT is not naturally occurring and comprises p73 residues 104-383.

[b] Prediction for specific genomes: Dm = *Drosophila melanogaster*, Dr = *Danio rerio*, Hs = *Homo sapiens*, Mm = *Mus musculus*, Xt = *Xenopus tropicalis.*

[c] Overlap with ΔNp63α
