# Supplementary material for: Conservation of DNA-binding specificity and oligomerisation properties within the p53 family
Source: BMC Genomics. 2009 Dec 23;10:628. doi: 10.1186/1471-2164-10-628 (PMC2807882; doi:10.1186/1471-2164-10-628)
Supplement: Additional file 8 — Figure S4. Sequence alignment of the DNA-binding domains of the p53 family members. [file 1471-2164-10-628-S8.PDF]

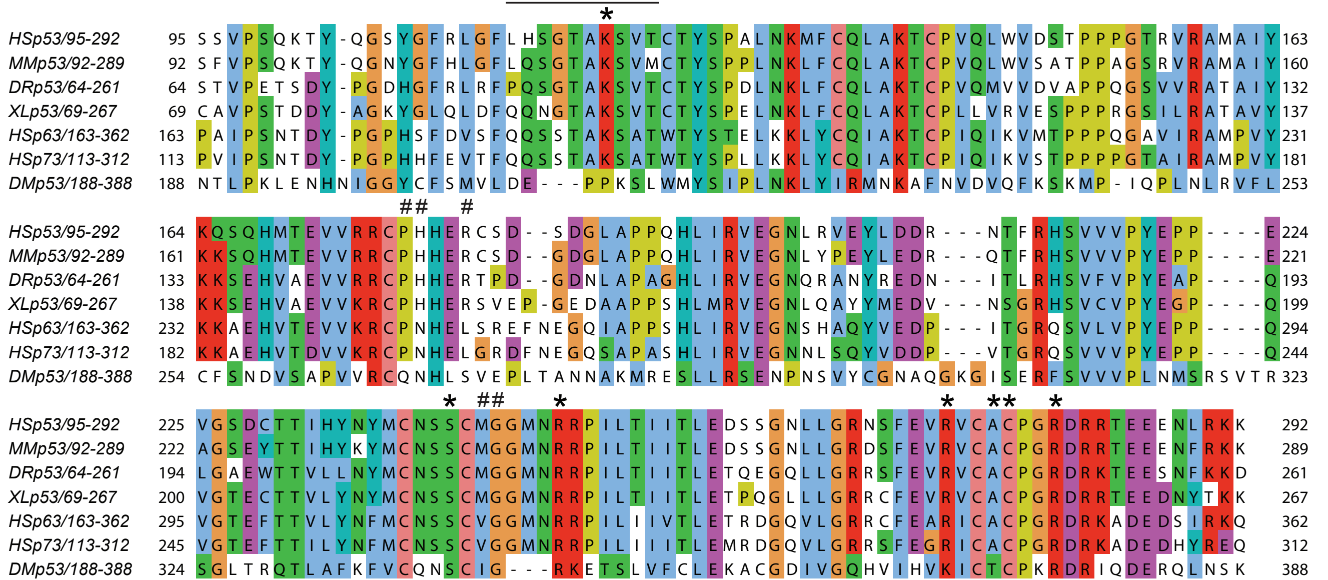

Figure S4. Sequence alignment of DNA-binding domains of p53 family members used in this study. DNA-contact residues in Hsp53 are marked with an asterisk, DBD interface residues with a hash. The flexible loop around the DNA-contact residue K120 is highlighted with a black bar. Colour coding is according to ClustalX [1].

1. Thompson JD, Gibson TJ, Plewniak F, Jeanmougin F, Higgins DG: The CLUSTAL_X windows interface: flexible strategies for multiple sequence alignment aided by quality analysis tools. Nucleic Acids Res 1997, 25(24):4876-4882.
