# Supplementary material for: Conservation of DNA-binding specificity and oligomerisation properties within the p53 family
Source: BMC Genomics. 2009 Dec 23;10:628. doi: 10.1186/1471-2164-10-628 (PMC2807882; doi:10.1186/1471-2164-10-628)
Supplement: Additional file 7 — Figure S3. Sequence alignment of the tetramerisation domains of the p53 family members. [file 1471-2164-10-628-S7.PDF]

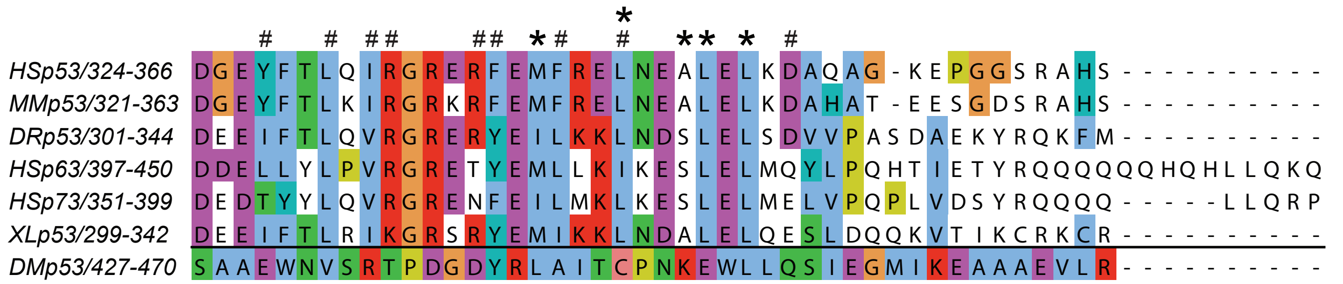

Figure S3. Sequence alignment of tetramerisation domains of p53 family members used in this study. Dimeric interface residues in Hsp53 are marked with a hash, tetrameric interface residues with an asterisk. Colour coding is according to ClustalX [1], where A,I,L,M,F,W,V – blue; R,K – red; N,Q,S,T - green; D,E - magenta; C - pink; G - orange; H,Y - teal; P - yellow, if residue-specific conservation criteria are met. The alignment was prepared using Jalview [2].

1. Thompson JD, Gibson TJ, Plewniak F, Jeanmougin F, Higgins DG: The CLUSTAL_X windows interface: flexible strategies for multiple sequence alignment aided by quality analysis tools. Nucleic Acids Res 1997, 25(24):4876-4882.

2. Waterhouse AM, Procter JB, Martin DM, Clamp M, Barton GJ: Jalview Version 2--a multiple sequence alignment editor and analysis workbench. Bioinformatics 2009, 25(9):1189-1191.
